# S100 Proteins in Alzheimer’s Disease

**DOI:** 10.3389/fnins.2019.00463

**Published:** 2019-05-16

**Authors:** Joana S. Cristóvão, Cláudio M. Gomes

**Affiliations:** ^1^Biosystems and Integrative Sciences Institute, Faculdade de Ciências, Universidade de Lisboa, Lisbon, Portugal; ^2^Departamento de Química e Bioquímica, Universidade de Lisboa, Lisbon, Portugal

**Keywords:** neuroinflammation and neurodegeneration, amyloid-β, tau, metal ions, protein misfolding and aggregation

## Abstract

S100 proteins are calcium-binding proteins that regulate several processes associated with Alzheimer’s disease (AD) but whose contribution and direct involvement in disease pathophysiology remains to be fully established. Due to neuroinflammation in AD patients, the levels of several S100 proteins are increased in the brain and some S100s play roles related to the processing of the amyloid precursor protein, regulation of amyloid beta peptide (Aβ) levels and Tau phosphorylation. S100 proteins are found associated with protein inclusions, either within plaques or as isolated S100-positive puncta, which suggests an active role in the formation of amyloid aggregates. Indeed, interactions between S100 proteins and aggregating Aβ indicate regulatory roles over the aggregation process, which may either delay or aggravate aggregation, depending on disease stage and relative S100 and Aβ levels. Additionally, S100s are also known to influence AD-related signaling pathways and levels of other cytokines. Recent evidence also suggests that metal-ligation by S100 proteins influences trace metal homeostasis in the brain, particularly of zinc, which is also a major deregulated process in AD. Altogether, this evidence strongly suggests a role of S100 proteins as key players in several AD-linked physiopathological processes, which we discuss in this review.

## Pathological Features of Alzheimer’s Disease

Alzheimer’s disease (AD) is a chronic and progressive neurodegenerative disorder that affects wide areas of the cerebral cortex and hippocampus. Most AD patients (>95%) are idiopathic and disease is characterized by late onset (80–90 years of age) with failure in the clearance of amyloid-β peptide (Aβ) from the brain (Masters et al., 2015). The main symptoms of the disease are progressive memory deficits, cognitive impairment, and personality changes. The neuropathological and neurochemical hallmarks of AD include selective neuronal death, synaptic loss and the presence of proteinaceous deposits in the extracellular space (known as diffuse and neuritic plaques) as well as inside neurons [known as neurofibrillary tangles (NFTs)]. Neuroinflammation, oxidative stress, and calcium dysregulation are also important features implicated in AD pathology (Wang X. et al., 2014).

Diffuse and neuritic plaques, most commonly known as amyloid plaques, are mainly constituted by Aβ deposits, surrounded by degenerative presynaptic endings, astrocytes and microglial cells (Weiner and Frenkel, 2006). Aβ peptides are formed from proteolytic cleavage of the amyloid precursor protein (APP) by the γ- and β-secretases (BACE1). Even though the normal function of APP is not known, it is possibly related to regulation of neurite outgrowth, cell adhesion, and neuron migration (Turner et al., 2003). APP processing can involve non-amyloidogenic or amyloidogenic pathways. When APP is cleaved by α-secretase and subsequently by γ-secretase, sAPPα is predominantly formed, which has an important role in neuronal plasticity and survival. However, in the amyloidogenic pathway, APP is cleaved by β- and γ-secretase producing sAPPβ, C-terminal fragments and Aβ peptides, which promote a range of detrimental effects in neurons and in the brain (Ling et al., 2003). Aβ40 and Aβ42 are the predominant accumulating peptides, whose aggregation into fibrillar cross-β structures is a central feature in AD pathogenesis. Aβ aggregation is naturally heterogeneous and monomers assemble and polymerize into structurally distinct forms, including protofibers, polymorphic oligomers and amyloid fibrils, all found within plaques. Extracellular accumulation of Aβ fibrils is not necessarily intrinsically cytotoxic and emerging evidence suggests precursor oligomers as the key toxic agents, also because of their seeding potential (Dahlgren et al., 2002; Walsh et al., 2002). Moreover, Aβ peptides can also be deposited intracellularly (Gouras et al., 2005).

The presence of neurofibrillar tangles, formed by neuronal intracellular deposition of hyperphosphorylated tau protein, is also a major AD hallmark. It has also been suggested that NFT may not be the major player in neurotoxicity, and that tau oligomers are in fact the major toxic forms promoting synaptic impairment (Tai et al., 2014; Fa et al., 2016). In agreement, it has been described that propagation of tau pathology occurs trans-synaptically (Liu et al., 2012). Other factors that contribute to tauophaties, besides tau hyperphosphorylation, are tau truncation, glycosylation, glycation, nitration, and ubiquitination (Chong et al., 2018).

Additionally, metal ion homeostasis and calcium signaling are also implicated in disease pathogenesis. In the early stages of AD, calcium imbalance promotes Aβ formation and tau hyperphosphorylation, as reviewed previously in LaFerla (2002). Aβ destabilizes neuronal calcium homeostasis generally leading to an increase in cytosolic calcium levels and formation of calcium-permeable pores. Calcium alterations lead to cytoskeletal modifications, triggering neuronal apoptosis and formation of free radicals through mitochondrial dysfunction. Moreover, familial AD mutations in presenilins are linked to altered synaptic Ca^2+^ signaling that imbalance the activities of Ca^2+^-calmodulin-dependent kinase II (CaMKII) and Ca^2+^-dependent phosphatase calcineurin (CaN), increasing the long-term depression and causing memory loss (Popugaeva et al., 2017). Transition metals such as Zn^2+^, Cu^2+^, and Fe^2+^ have well-established roles as chemical modulators of protein folding, amyloid aggregation and toxicity and are found to accumulate at protein deposits (Leal et al., 2012; Barnham and Bush, 2014; Cristovao et al., 2016).

Neuroinflammation is another cellular process linked to AD pathogenesis. Senile plaques are often closely associated with activated microglial cells and surrounded by activated astrocytes that have abundant filaments (Weiner and Frenkel, 2006). In response to Aβ deposition, activated microglia upregulate the expression of cell-surface proteins and cytokines such as the tumor-necrosis factor (TNF), interleukine-6 (IL-6), interleukine-1 (IL-1), S100 proteins, and chemokines. The presence of Aβ activates different cell receptors and intracellular signaling pathways, mainly those related to the receptor of advanced glycation end products (RAGE)/nuclear factor kappa-light-chain-enhancer of activated B cells (NF-κB) pathway, that is responsible for the transcription of pro-inflammatory cytokines and chemokines in astrocytes (Gonzalez-Reyes et al., 2017). Additionally, astrogliosis appears as an early manifestation of AD. The migration of astrocytes into Aβ plaques is promoted by chemokines CCL2 and CCL3, that are released by activated microglial cells surrounding amyloid plaques. Astrocytes recruited to Aβ plaques have the potential to mediate both neurotoxicity and participate in the clearance of Aβ (Weiner and Frenkel, 2006). S100 proteins are among the alarmins that are upregulated and are highly secreted by astrocytes during this process (Venegas and Heneka, 2017), which results in their accumulation within Aβ deposits and brain tissues, as overviewed in the following sections.

## The S100 Protein Family

S100 proteins are a family of low-molecular weight EF-hand Ca^2+^ proteins that are expressed in distinct organs and tissues. They are involved in multiple intracellular functions, including cell proliferation, differentiation, protein phosphorylation, cytoskeletal assembly, and disassembly and intracellular calcium homeostasis (Mrak and Griffinbc, 2001; Donato et al., 2009, 2013). In pathological conditions S100 proteins can be expressed in a cell type where they are not expressed under normal conditions. Additionally, some S100 proteins are secreted and regulate cell functions in an autocrine or paracrine manner by activation of surface receptors, such as the RAGE receptor, thereby promoting NF-κB signaling, an important trigger of inflammatory processes, recruiting and activating cellular pro-inflammatory effectors (Hofmann et al., 1999; Leclerc et al., 2009). Albeit S100 proteins are not cytokines in *stricto sensu*, in these cases, they have such functions, and act as extracellular alarmins or as damage-associated molecular pattern (DAMP) factors, that can either be beneficial or detrimental depending on concentration and molecular and cellular moiety (Donato et al., 2009, 2013). From the 25 S100 proteins described so far, several are present in the brain and from those, seven have been implicated in AD pathways: S100B, S100A1, S100A6, S100A7, S100A8, S100A9, and S100A12.

S100 proteins occur mainly as homodimers (Barger et al., 1992; Giannakopoulos et al., 1996; Matsui Lee et al., 2000; Cunden et al., 2017). Specifically, it is known that calcium binding to S100 proteins triggers conformational changes that expose a hydrophobic cleft that is crucial to interaction with partners to their activation, regulation and signaling functions. Several S100 proteins also bind zinc and copper, which interestingly are highly abundant in senile plaques (Heizmann et al., 2002; Senior et al., 2003; Maynard et al., 2005). A few S100 proteins are also found as heterodimers, including S100A8/A9 (Teigelkamp et al., 1991), S100B/A1 (Garbuglia et al., 1999) and S100A6/B (Yang et al., 1999). S100 proteins interconvert into functional oligomers, including tetramers, hexamers, and octamers and formation of these species can be promoted by Ca^2+^ or Zn^2+^ binding (Botelho et al., 2012). The functions described for these S100 oligomers involve a tighter interaction with RAGE, assistance in microtubule formation, neurite outgrowth, and tumor suppression.

S100 proteins influence cognitive processes in the healthy brain and play roles in development and neuronal maintenance. Depending on the study, antisera to neurospecific S100 protein (Gromov et al., 1992; O’Dowd et al., 1997; Epstein et al., 2006) and antibodies directed against S100A1 and S100B (Gromov et al., 1992; O’Dowd et al., 1997; Epstein et al., 2006) either impair or improve learning and memory functions in rat brains (Gromov et al., 1992; O’Dowd et al., 1997; Epstein et al., 2006). Recent studies suggested that elevated S100B levels have deleterious effects during the neurodevelopmental period through RAGE-dependent processes (Santos et al., 2018). Additionally, S100B has been associated with Down Syndrome, a genetic variation where the most profound neurological features are mental retardation, seizures and early onset AD. Levels of S100B are increased in neuronal progenitor cells of patients with Down Syndrome (Esposito et al., 2008a) and in human induced pluripotent stem cells derived from Down Syndrome patients (Chen et al., 2014). S100B overexpression causes toxicity to neurons, reduces neurogenesis, and increases the production of reactive oxygen species (Esposito et al., 2008a; Lu et al., 2011; Chen et al., 2014).

There are several studies using clinical specimens and animal models implicating S100 proteins in AD pathophysiology (Marshak et al., 1992; Akiyama et al., 1994; Sheng et al., 1994; Gerlai et al., 1995; Mrak et al., 1996; Boom et al., 2004; Shepherd et al., 2006; Chaves et al., 2010; Ha et al., 2010; Mori et al., 2010; Roltsch et al., 2010; Chang et al., 2012; Afanador et al., 2014; Cirillo et al., 2015; Horvath et al., 2016; Gruden et al., 2017; Lodeiro et al., 2017; Iashchishyn et al., 2018; Wang et al., 2018). As overviewed in the following sections, different S100 proteins seem to be involved in several processes related to APP processing, influencing Aβ levels, tau post-translational modifications, formation of protein inclusions, and multiple signaling pathways. The ability of acting as Ca^2+^ sensors, regulating the activity of channels/pumps involved in Ca^2+^ release/uptake also provide feedback and feedforward mechanisms for sustaining aberrant Ca^2+^ signaling in AD. Therefore, involvement in all these processes makes a strong case for the importance of the S100 protein family in AD development (Table 1).

**Table 1:** Distribution, levels, and implication of brain S100 proteins in AD pathways.

	S100A1	S100A6	S100A7	S100A8	S100A9	S100A12	S100B
Expression	Neurons (Isobe et al., 1984)	Astrocytes (Boom et al., 2004; Yamada and Jinno, 2012); Neurons (Yamada and Jinno, 2014)	Neurons (Qin et al., 2009)	Microglia (Akiyama et al., 1994; Kummer et al., 2012); Neurons (Wang et al., 2018)	Microglia (Akiyama et al., 1994; Kummer et al., 2012); Neurons (Shepherd et al., 2006; Wang C. et al., 2014)	Neurons (Shepherd et al., 2006); Glia (Shepherd et al., 2006)	Astrocytes (Van Eldik and Griffin, 1994; Mrak et al., 1996; Shepherd et al., 2006); Oligodendrocytes (Shepherd et al., 2006); Neurons (Yang et al., 1995; Ichikawa et al., 1997); Microglia (Adami et al., 2001).

APP processing	n/a	n/a	- S100A7 ↑ α-secretase activity via ADAM-10 (Qin et al., 2009).	n/a	- S100A9 knockdown in Tg2576 ↓APP-CT (Chang et al., 2012); - S100A9 knockout in APP/PS1 transgenic mice ↓ BACE expression and activity (Kummer et al., 2012); - S100A9 knockdown in Tg2576 ↑neprilysin and ↓BACE activity (Chang et al., 2012). - Inhibitor of γ-Secretase ↓ S100A9 in BV2 cells (Li et al., 2014). - C-terminal fragments of APLP2 ↑ S100A9 in BV2 cells (Li et al., 2014).	n/a	- S100B ↑APP levels in rat retinal neurons (Anderson et al., 2009); - Tg2576-huS100B mice ↑ soluble APPβ and ↑BACE1 (Mori et al., 2010).

Aβ levels	n/a	- Exogenous S100A6 treatment ↓Aβ levels (Tian et al., 2019)	- S100A7 inhibits Aβ42 and Aβ40 generation in primary neurons from Tg2576 transgenic embryos (Qin et al., 2009).	- Treatment with Aβ ↑S100A8 in glia and astrocytes (Lodeiro et al., 2017); - Treatment of SH-SY5Y cells with S100A8: ↑Aβ42 and ↓Aβ40 production (Lodeiro et al., 2017); - Aβ ↑S100A8 mRNA expression (Walker et al., 2006); - S100A8/A9 interacts with Aβ40 (ESI-MS) (Lee et al., 2018); - S100A8/A9 ↓Aβ40 amyloid level (Lee et al., 2018).	- Aβ induces S100A9 expression in the microglial cell line BV-2 (Ha et al., 2010); - S100A9 knockdown in Tg2576 ↓Aβ (Chang et al., 2012); - Aβ42 monomers ↓release of S100A9 in human THP-1 monocytes (Lee et al., 2013); - S100A9 interacts with Aβ40 and promotes the formation of amyloid structures (Zhang et al., 2012; Zhao et al., 2013); - S100A9 interacts with Aβ40 (NMR) (Wang C. et al., 2014); - Cytotoxicity of S100A9 is suppressed by Aβ40 (Zhang et al., 2012); - Coaggregation of S100A9 with Aβ40 and Aβ42 (Wang C. et al., 2014).	n/a	- Aβ injection on rat retinal neurons ↑S100B expression (Anderson et al., 2009); - Overexpressing S100B in Tg2576 mice ↑Aβ levels and amyloid deposits (Mori et al., 2010); - Nanomolar concentration of S100B protect against Aβ-mediated cytotoxicity (Businaro et al., 2006; Clementi et al., 2013, 2016); - S100B interacts with Aβ42 (NMR, ITC, SAXS) (Cristovao et al., 2018); - S100B suppresses Aβ42 aggregation and cellular toxicity in a calcium-tuned manner (Cristovao et al., 2018).

Amyloid plaques	- S100A1 knockout in PS/APP mice ↓plaque load (cortical and hippocampal regions) (Afanador et al., 2014); - S100A1 in amyloid plaques of murine and human AD specimens (Afanador et al., 2014).	- Associated with amyloid plaques (Boom et al., 2004; Tian et al., 2019); - Exogenous S100A6 treatment ↓plaque burden (Tian et al., 2019) - S100A6 is co-localized with S100B and GFAP near amyloid plaques (Boom et al., 2004).	n/a	- S100A8 aggregates observed prior to formation of Aβ plaques (Lodeiro et al., 2017); - ↑S100A8 in microglial cells around amyloid plaques (Kummer et al., 2012);	- Associated with amyloid plaques (Shepherd et al., 2006); - ↑S100A9 in microglial cells around amyloid plaques (Kummer et al., 2012); - S100A9 knockout ↑phagocytosis of fibrillar amyloids in microglia cells and ↓Aβ deposition (Kummer et al., 2012); - Isolated plaques of S100A9 and Aβ (Horvath et al., 2016); - S100A9 knockdown ↓amyloid plaque burden (Ha et al., 2010).	- Associated with amyloid plaques (Shepherd et al., 2006).	- Associated with amyloid plaques (Shepherd et al., 2006); - Present in diffuse (non-neuritic) amyloid deposits (Mrak et al., 1996); - Overexpression of S100B ↑large plaques (Mori et al., 2010); - PSAPP/S100B^-/-^↓cortical amyloid plaque load and number (Roltsch et al., 2010); - S100B-positive astrocytes surround neuritic plaques (Van Eldik and Griffin, 1994).

Tau	- Ablation of S100A1 expression ↑tubulin/ microtubules levels in PC12 cells (Zimmer et al., 1998); - S100A1 causes disassembly of microtubules in U251 glioma cells and rat L6 myoblasts (Sorci et al., 2000).	- ↑S100A6 interferes with CacyBP/SIP complex and inhibits its activity and ↓Tau dephosphorylation in NB2a cells (Wasik et al., 2013).	n/a	n/a	- Associated with neurons with neurofibrillary-tangle morphology (Shepherd et al., 2006).	n/a	- ↑ S100B leads to hyperphosphorylated Tau in human neural stem cells (Esposito et al., 2008b); - DKK-1 inhibition abolish S100B-induced tau hyperphosphorylation (Esposito et al., 2008b); - S100B binds Tau through kinase II and inhibits Tau phosphorylation (Baudier and Cole, 1988); - S100B levels are correlated to Tau plaques (Sheng et al., 1994); - Neurofibrillar tangles of parahippocampal cortex of AD patients are correlated to S100B positive astrocytes (Sheng et al., 1997); - PSAPP/S100B ^-/-^↓phospho-tau positive dystrophic neurons (Roltsch et al., 2010); - S100B causes disassembly of microtubules in U251 glioma cells and rat L6 myoblasts (Sorci et al., 2000).

CSF levels	n/a	n/a	- ↑ S100A7 in AD patients (Qin et al., 2009).	n/a	- ↓S100A9 and Aβ42 levels in AD patients (Horvath et al., 2016).	n/a	- ↑ S100B in AD patients (Petzold et al., 2003); - ↑ S100B in mild/moderate AD patients (Peskind et al., 2001).

Inflammation	- S100A1 knockout in PSAPP AD mouse ↓ astrocytosis, microgliosis (Afanador et al., 2014).	n/a	n/a	n/a	n/a	n/a	- Astrocytosis and neurite proliferation in transgenic mice expressing elevated levels of S100B (Reeves et al., 1994); - Astrocytosis and microgliosis in Tg2576 mice overexpressing S100B (Mori et al., 2010); - PSAPP/S100B ^-/-^↓cortical gliosis (Roltsch et al., 2010); - S100B inhibitor ↓ reactive gliosis, ↓ astrocyte infiltration and rescues neuronal loss in Aβ-injected mice (Cirillo et al., 2015).

Signaling pathways	- S100A1 inhibits Akt/GS3β signaling (Afanador et al., 2014).		- S100A7 promotes Erk1/2 and PKC phosphorylation (Qin et al., 2009).	n/a		n/a	- 3XTg-AD mice with an IL-1 inhibitor ↓S100B levels and suppress Wnt/β-catenin (Kitazawa et al., 2011); - ↑ IL-1β and IL-6 mRNA expression in Tg2576-huS100B mice (Mori et al., 2010); - ↑ S100B activates JNK, degrades β-catenin, and disrupts Wnt pathway in human neural stem cells (Esposito et al., 2008b); - Inhibition of S100B causes ↓ GFAP,↓ p-p38 MAPK, ↓COX-2, ↓ IL-1β and ↓RAGE expression in C57BL/6J mice (Cirillo et al., 2015); - TNFα ↓S100B expression in astrocytes and ↑S100B extracellular levels in primary astrocytes (Edwards and Robinson, 2006).

S100 conformers	Found in extracellular deposits (Afanador et al., 2014).	Found in clusters around amyloid plaques (Boom et al., 2004).	n/a	Found isolated S100A8 clusters in the hippocampi of Tg2576 and TgAPPartic AD mice models (Lodeiro et al., 2017).	- Found as dimers and as S100A9 multimers in AD brain patients (Shepherd et al., 2006); - Found isolated S100A9 clusters in AD brain tissues (Horvath et al., 2016; Wang et al., 2018).	- Found hexameric S100A12 in AD brain patients (Shepherd et al., 2006).	- Found as native dimers and as higher order multimers in AD brain patients (Shepherd et al., 2006); - Found isolated S100B clusters around tau plaques (Sheng et al., 1994; Mrak et al., 1996).

Brain region	n/a	- ↑ S100A6 in white matter; in gray matter is concentrated in amyloid plaques of AD patients (Boom et al., 2004); - S100A6 in amygdala and hippocampus in APP/London mice (Boom et al., 2004); - ↑ S100A6 expression in APP/PS1KI mice (Wirths et al., 2010; Weissmann et al., 2016).	- ↑ S100A7 in amygdala and hippocampus of AD brain patients (Qin et al., 2009); - ↑ S100A7 in serum of mild cognitively impaired patients (Mueller et al., 2010).	- ↑ S100A8 in hippocampus of Tg2576 and TgAPPartic mice (Lodeiro et al., 2017); - ↑ S100A8 in serum of AD patients (Shen et al., 2017).	- ↑S100A9 in familial and sporadic AD patients (Shepherd et al., 2006); - ↑S100A9 expression in brain lysates of AD patients (Kummer et al., 2012); - ↑S100A9 expression in Tg2576 mice and AD patients (Chang et al., 2012); - ↑S100A9 expression in cortex and hippocampus of CT-Tg and Tg2576 mice model and in AD brain patients (Ha et al., 2010); - S100A9 in plaques of hippocampal and neocortical areas of AD patents in Braak stages III to VI (Wang C. et al., 2014).	n/a	- S100B in cortical and white matter of PS-1 and sporadic AD brains (Shepherd et al., 2006); - S100B in layer I cortex of AD brains (Simpson et al., 2010); - ↑ S100B hippocampus, temporal lobe, frontal lobe and pons in AD brains (Van Eldik and Griffin, 1994).


*n/a, no data available.*

### S100B

S100B is the most studied S100 protein in the scope of AD, as reviewed in Steiner et al. (2011) and Yardan et al. (2011). S100B acts as a pro-inflammatory cytokine and a DAMP molecule depending on its concentration. S100B secreted from astrocytes can have trophic and toxic effect on neurons. At nanomolar concentration, S100B displays neurotrophic effects, leading to promotion of neuronal survival and neurite outgrowth. At micromolar concentrations S100B has deleterious effects inducing neuronal apoptosis (Mrak and Griffinbc, 2001). Upregulation of S100B leads to behavioral abnormalities and loss of dendritic density in mice (Gerlai et al., 1995). S100B also regulates the intracellular levels of free calcium in several central nervous system cell types, such as neurons and astrocytes. Recently we demonstrated that S100B acts as a sensor and regulator of elevated zinc levels in the brain and that this metal-buffering activity is tied to a neuroprotective role, through an indirect effect on calcium levels and in inhibition of excitotoxicity (Hagmeyer et al., 2017).

Several studies point to high levels of S100B in AD patients (Marshak et al., 1992; Van Eldik and Griffin, 1994; Peskind et al., 2001; Petzold et al., 2003; Chaves et al., 2010) and in AD mouse models (Yeh et al., 2015). The largest increase in S100B levels is observed in the hippocampus, temporal lobe (Van Eldik and Griffin, 1994) and in the layer I of the cortex (Simpson et al., 2010). It is demonstrated that sera of AD patients with moderate and severe dementia have a 60- and 37-fold increase in S100B, respectively. In AD patients with moderate dementia, the increase of S100B levels is followed by a 10-fold increase in auto-antibodies: however, in AD patients with severe dementia the levels of auto-antibodies remain identical to controls (Gruden et al., 2007), indicating that there is no immune-protection against elevated S100B levels in AD patients with severe dementia. S100B is also elevated in the cerebrospinal fluid (CSF) of AD patients (Peskind et al., 2001; Petzold et al., 2003). S100B is involved in APP cleavage processes: high levels of S100B increase BACE1 activity resulting in higher levels of toxic APPβ and C-terminal fragments, including the amyloidogenic β-CTF (C99) (Anderson et al., 2009; Mori et al., 2010).

S100B surrounding amyloid plaques is mostly produced by astrocytes (Van Eldik and Griffin, 1994; Mrak et al., 1996; Shepherd et al., 2006; Roltsch et al., 2010), but can also originate from oligodendrocytes (Simpson et al., 2010) and microglia (Roltsch et al., 2010). It was also observed that S100B positive astrocytes are present in the diffuse non-neuritic amyloid plaques (Mrak et al., 1996), suggesting an early, yet unclear, action of S100B in the formation of senile plaques. Evidence suggests that S100B may regulate plaque formation as the knockout of S100B in the PS/APP AD mouse model selectively decreases plaque load in the cortical region (Roltsch et al., 2010) and the overexpression of S100B increases Aβ levels and deposits, at early stages (Mori et al., 2010). Even though, it is established that elevated levels of S100B have deleterious effects that promote AD features, nanomolar concentrations of S100B effectively protect cells against Aβ-mediated cytotoxicity (Businaro et al., 2006; Clementi et al., 2013, 2016). Additionally, we have recently found that, *in vitro*, S100B binds to Aβ42 monomers, oligomers, and fibrils resulting in a calcium-tuned suppression of Aβ42 aggregation and cellular toxicity in SH-SY5Y cells (Cristovao et al., 2018). S100B was found both in normal and in AD brains in various oligomeric states (Shepherd et al., 2006); however, the protective or pathological functions of S100B oligomers are still unclear.

Overexpression of S100B in Tg2576 AD transgenic mice is also linked with neuroinflammation, promoting astrogliosis, microgliosis, and neurite proliferation (Reeves et al., 1994; Mori et al., 2010). However, knockout of S100B in PS/APP mouse model decreases cortical gliosis (Roltsch et al., 2010). IL-1 regulates the expression and secretion of S100B from astrocytes (de Souza et al., 2009). Treatment of the 3XTg-AD mice with an antibody against IL-1 reduces S100B levels and results in attenuation of tau pathology and in partial reduction of certain fibrillar and oligomeric forms of Aβ (Kitazawa et al., 2011) Therefore, S100B seems to be tied to different processes related to AD pathology as in addition to its ability to promote brain inflammatory response and tau pathology (Esposito et al., 2008b) it may play roles in directly promoting amyloidogenic APP processing, as proposed by Mori et al. (2010).

Inhibition of S100B using pentamidine in AD mouse models, lead to a reduction in the levels of proinflammatory mediators such as nitrite, MDA, PGE2 and IL-1, followed by an inhibition of Aβ-induced gliosis (Cirillo et al., 2015). Indeed, S100B-overexpressing mice that were infused with oligomeric Aβ exhibited enhanced glial activation. Neuroinflammation and loss of synaptic markers were noted, however there was no difference in the amyloid plaque burden, in comparison to controls (Craft et al., 2005). These results suggest a relationship between S100B and other cytokines that are also implicated in AD pathways. Indeed, the TNF-α cytokine, which is present at high levels in the AD brain, decreases both GFAP and S100B intracellular levels in astrocytes, while increasing their extracellular levels (Edwards and Robinson, 2006). This crosstalk suggests a relationship between TNF-α and the increase of these two proteins in CSF and sera of AD patients. Other reports showed that S100B enhances IL-6 mRNA (Mori et al., 2010; Yeh et al., 2015) and IL-1 mRNA levels in microglia and in neurons (Mori et al., 2010), via Sp1 and NF-κB signaling pathways (Liu et al., 2005). Additionally, knockout of S100B in the Tg2576 AD mouse model background results in a decrease in GFAP-positive astrocytes and in Iba-1 positive microglia (Roltsch et al., 2010), while its overexpression has opposite effects (Mori et al., 2010). Overall, these results suggest that S100B can influence and be influenced by the levels of other cytokines involved in AD pathogenesis.

Regarding tau pathology, high S100B levels in AD patients positively correlate to tau tangles with which S100B was found to be clustered (Sheng et al., 1994, 1997). Knockout of S100B in the PS/APP mouse model decreases phosphorylated-tau positive dystrophic neurons (Roltsch et al., 2010) and in mouse models expressing tau, S100B levels are upregulated (Sidoryk-Wegrzynowicz et al., 2017). Indeed, it has been demonstrated, *in vitro*, that S100B directly binds tau inhibiting its phosphorylation by yet unclear non-covalent interactions in a process that is Ca^2+^ or Zn^2+^-dependent (Baudier and Cole, 1988). However, contradictory results show that S100B promotes tau hyperphosphorylation by inducing GSK-3β activation and disrupting Wnt signaling (Esposito et al., 2008b), an important pathway to regulate synaptic transmission and plasticity. Indeed, S100B promotes the expression of the Dickkopf-related protein 1 (Dkk1), an antagonist of Wnt signaling that has previously been suggested to play a role in AD (Guo et al., 2016).

As previously mentioned, calcium dysregulation contributes to AD pathology and S100B is a key factor in the Ca^2+^ homeostasis of astrocytes. It was demonstrated that S100B knockout leads to a decrease of induced-Ca^2+^ transients (Xiong et al., 2000) such as those induced by Aβ. In what could be a potentially protective mechanism, S100B levels were found to be up-regulated in astrocytes upon Aβ induced Ca^2+^ intracellular waves (Chow et al., 2010).

### S100A1

The investigation of the role of S100A1 in AD is encouraged by the fact that some of its targets are altered in the disease, such as the ryanodine receptor (RyR), an intracellular calcium release channel, tau and RAGE. S100A1 is primarily expressed in neurons and, as reviewed in Zimmer et al. (2005) is implicated in tau phosphorylation, neuronal cell sensitivity to Aβ and in the regulation of APP expression. In respect to the latter, available data indicates that βAPP steady-state mRNA and intracellular protein levels are down-regulated in response to ablation of S100A1 expression (Zimmer et al., 2005). In the PS/APP mouse model, knockout of S100A1 decreases inflammatory processes, such as astrocytosis and microgliosis, diminishing 3.7-fold the number of cortical plaques and 1.5-fold the number of hippocampal plaques (Afanador et al., 2014). Decreased S100A1 levels in PC12 cells increase tubulin levels and the number of neurites (Zimmer et al., 1998). Additionally, knockout of S100A1 in PC12 cells increases the resistance to Aβ-induced cell death (Zimmer et al., 2005).

S100A1 induces Glycogen synthase kinase 3 (GSK3) phosphorylation (Afanador et al., 2014), that is involved in several processes such as glycogen metabolism and gene transcription. GSK3 over-activation is also related to memory impairment and other AD related features (Hooper et al., 2008). In human and mouse AD brain tissue, S100A1:RyR complexes are present and their formation is Ca^2+^-dependent. RyR is a receptor with altered levels in AD that is associated with APP processing and Aβ production, however it is not known if it exerts a protective or pathogenic role in AD (Del Prete et al., 2014). S100A1 also binds to stress-inducible phosphoprotein 1 (STIP1) (Maciejewski et al., 2017), a Hsp90 cochaperone that is reported to be present in the vicinity of Aβ oligomers, preventing Aβ-induced synaptic loss and neuronal death in primary neurons (Ostapchenko et al., 2013). S100A1 and S100B also have the ability to cause microtubule disassembly in glioma cells and myoblasts in a Ca^2+^-dependent manner, suggesting a possible role of S100A1 in tau pathology (Sorci et al., 2000). Moreover, in human AD patients and in the PS/APP mouse model, extracellular S100A1 has been observed in plaque-like deposits (Afanador et al., 2014).

### S100A6

S100A6 was identified in the AD gene signature as one of the most significantly positively correlated proteins with AD phenotype (Wruck et al., 2016). As other S100 proteins, S100A6 is upregulated in AD patients and in AD mouse models (Boom et al., 2004; Wirths et al., 2010; Weissmann et al., 2016) and is found in astrocyte-positive clusters that surround Aβ amyloid deposits in the brain’s gray matter (Boom et al., 2004). In PS/APP mouse brains, S100A6 localizes in the peripheral region of amyloid plaques and exogenous S100A6 treatment in mouse brain sections reduces Aβ levels and plaque burden (Tian et al., 2019).

Zinc ions are colocalized with senile plaques in AD patients and there is evidence that AD related-cognitive decline is dependent on extracellular zinc levels (Lovell et al., 1998; Takeda and Tamano, 2016). In particular, one study suggested that zinc-binding S100A6 exerts a zinc sequestering function, identical to what has been proposed for S100B (Hagmeyer et al., 2017), thus preventing zinc-induced toxicity in COS-7 cells (Tian et al., 2019). Additionally, PS/APP mice treated with a high-zinc diet have increased S100A6 levels and Aβ deposits. These studies point to a correlation between S100A6, zinc ions and decrease in Aβ plaque load.

The heterodimer S100A6/B is also implicated in pathological signal transduction in melanoma (Yang et al., 1999). It is possible that the formation of the heterodimer also occurs in AD since S100A6 is colocalized with S100B and astrocytic glial fibrillary acidic protein (GFAP), a marker of astrogliosis, near amyloid plaques (Boom et al., 2004). Additionally, it is reported that S100A6 binds to the CacyBP/SIP complex, a complex known to participate in the organization of microtubules. Overexpression of S100A6 in neuroblastoma NB2a cells inhibits CacyBP/SIP complex activity, and consequently lowers the rate of tau dephosphorylation (Wasik et al., 2013).

### S100A7

There is scarce evidence regarding the role of S100A7 on AD pathways. It is reported that S100A7 is increased in mildly cognitively impaired patients (Mueller et al., 2010) and in the brain and CSF of AD dementia patients (Qin et al., 2009). S100A7 mRNA expression is regulated in the brain as a function of AD dementia and amyloid neuropathology (Qin et al., 2009). Exogenous S100A7 in primary hippocampal neurons of Tg2576 AD transgenic embryos inhibits the generation of Aβ42 and Aβ40 peptides and promotes the activity of “non- amyloidogenic” α-secretase, via upregulation of ADAM-10 (a disintegrin and metalloproteinase) and phosphorylation of Erk1/2 and PKC (Qin et al., 2009). Therefore, a beneficial role of S100A7 on APP processing is suggested, albeit other studies are required to more extensively support this possibility.

### S100A8

S100A8 was found to be upregulated in the sera of AD patients (Shen et al., 2017) and in the hippocampus of Tg2576 and TgAPParc AD mice (Lodeiro et al., 2017). Indeed, several studies establish a correlation between Aβ and S100A8 expression. An increase in S100A8 mRNA levels was induced when aggregated Aβ was added to a microglia culture isolated from post-mortem AD brain tissues. Subsequent culture growth suggested that chronic secretion of S100A8 can lead to chronic activation of microglia (Walker et al., 2006). In rat primary astrocytes, Aβ42 treatment induces a significant increase in S100A8 mRNA levels. Treatment of SH-SY5Y neuroblastoma cells with recombinant S100A8 increased Aβ42 and decreased Aβ40 production (Lodeiro et al., 2017). In PS/APP mice, the S100A8/A9 heterodimer is found to be upregulated in microglial cells surrounding amyloid plaques (Kummer et al., 2012). Additionally, it is reported that S100A8/A9 binds directly to Aβ40 and that it interferes with amyloid formation but no effect was observed over Aβ42 aggregation (Lee et al., 2018). There is also a link between S100A8/A9 and the “non-amyloidogenic” α-secretase ADAM-10, since S100A8/A9 has lower expression in AD mice models overexpressing ADAM-10 (Prinzen et al., 2009).

S100A8 assemblies were found in the hippocampus of Tg2576 and TgAPParc AD mice brains, distinct from corpora amylacea, that are formed independently of Aβ plaques (Lodeiro et al., 2017). These S100A8 aggregates are likely not amyloidogenic as no staining was observed with thioflavin-S (Lodeiro et al., 2017).

### S100A9

S100A9 was found to be strongly increased in brain lysates of AD patients and AD mice compared to healthy, age-matched controls (Ha et al., 2010; Chang et al., 2012; Kummer et al., 2012) and in familial PS-1 AD tissues (Shepherd et al., 2006). S100A9 is present in activated glia and neurons positive for tau neurofibrillary tangles (Shepherd et al., 2006). Additionally, there is a strong correlation between S100A9 and Aβ. In *in vitro* cell assays, Aβ42 reduces extracellular release of S100A9 in human THP-1 monocytes (Lee et al., 2013) and induces S100A9 expression in microglia BV2 cells (Ha et al., 2010). However, in CSF from AD patients with mild cognitive impairment and vascular dementia, the levels of S100A9 and Aβ42 are decreased (Horvath et al., 2016). Knockdown of S100A9 decreases cognition decline on Tg2576 mice and reduces amyloid plaque burden (Ha et al., 2010; Chang et al., 2012). S100A9 was found within amyloid plaques of sporadic and familial PS-1 AD brains (Shepherd et al., 2006; Wang et al., 2018) with distinct Braak stages from III to VI (Kummer et al., 2012; Wang C. et al., 2014). Indeed, in some studies it was possible to observe Aβ42 plaques and also isolated S100A9 plaques that are not colocalized, forming separate tissue deposits (Horvath et al., 2016; Wang et al., 2018).

Regarding the formation of S100A9 puncta in AD brain, a recent study reported that, *in vitro*, S100A9 is able to form polymeric structures that resemble amyloid structures and bind amyloid fluorophores. The polymerization reaction occurs through a two-step nucleation with initial misfolding of S100A9 and β-sheet formation (Iashchishyn et al., 2017). The impact of S100A9 oligomers on memory was studied through intranasal administration of S100A9 (dimers, oligomers, and fibrillar states) in aged mice. S100A9 oligomers and fibrils, but not dimers, evoked amnestic activity which is correlated with disruption of dopaminergic and glutamate neurochemistry in the prefrontal cortical and hippocampal regions (Gruden et al., 2016, 2018). Additionally, intranasal administration of S100A9 induces cellular stress in the frontal lobe, hippocampus, and cerebellum of aged mice, as well as impaired learning. However, co-treatment with S100A9 fibrillar species and glutamate antibodies increases locomotor activity (Gruden et al., 2017; Iashchishyn et al., 2018). In Tg2576 AD mice, knockdown or knockout of S100A9 significantly reduced the neuropathology and greatly improved learning and memory (Chang et al., 2012), suggesting a link between S100A9 and AD pathology. Indeed, knockout of S100A9 in the PS/APP AD mouse model led to increased phagocytosis of fibrillar Aβ and to decreased Aβ deposition (Kummer et al., 2012).

However, several studies focused on the relationship between S100A9 and Aβ42. *In* *vitro* biophysical approaches showed that S100A9 binds to Aβ40 through hydrophobic interactions (Zhang et al., 2012; Zhao et al., 2013; Wang C. et al., 2014). Kinetic assays suggested that S100A9 co-aggregates with Aβ40, promoting the formation of amyloid fibrils. The co-aggregation of S100A9 with Aβ42 was also referred to inhibit Aβ42 cytotoxicity (Wang C. et al., 2014).

Regarding APP processing, it was found that C-terminal fragments of amyloid precursor-like protein 2 (APLP2) upregulate S100A9 protein and mRNA expression in BV2 cell and that inhibitor of γ-secretase promotes downregulation of S100A9 protein levels (Li et al., 2014). AD mice deficient in S100A9 have decreased levels of key cytokines involved in APP processing and a reduction of BACE1 expression and activity (Kummer et al., 2012). S100A9 knockout also reduced overall levels of Aβ and APP C-terminal fragments in Tg2576 AD mice, due to increase in neprilysin levels and decreased BACE activity (Chang et al., 2012). Knockdown of S100A9 significantly attenuated the increase of Ca^2+^ levels provoked by C-terminals of APP or by Aβ treatment (Ha et al., 2010); however, others observed that a reduction of S100A9 extracellular release is followed by an increase in intracellular Ca^2+^ levels (Lee et al., 2013), evidencing a correlation between S100A9 and calcium dysregulation in AD. In the AD brain and in mouse models, S100A9 is present in its native form but also as large complexes ranging from 90 to 190 kDa (Shepherd et al., 2006). Indeed, after intranasal administration of S100A9 fibrils in aged mice, S100A9 plaques were observed in the brain which resulted in an exacerbation of cell stress (Iashchishyn et al., 2018). Overall there are solid evidences regarding S100A9 as a potential regulator of AD pathways.

### S100A12

S100A12 is the least studied S100 protein in the context of AD. To date, a single study reported S100A12 hexamers associated with senile plaques, reactive glia and neurons in brains of sporadic and PS-1 AD patients (Shepherd et al., 2006).

## Outlook

**FIGURE 1:**
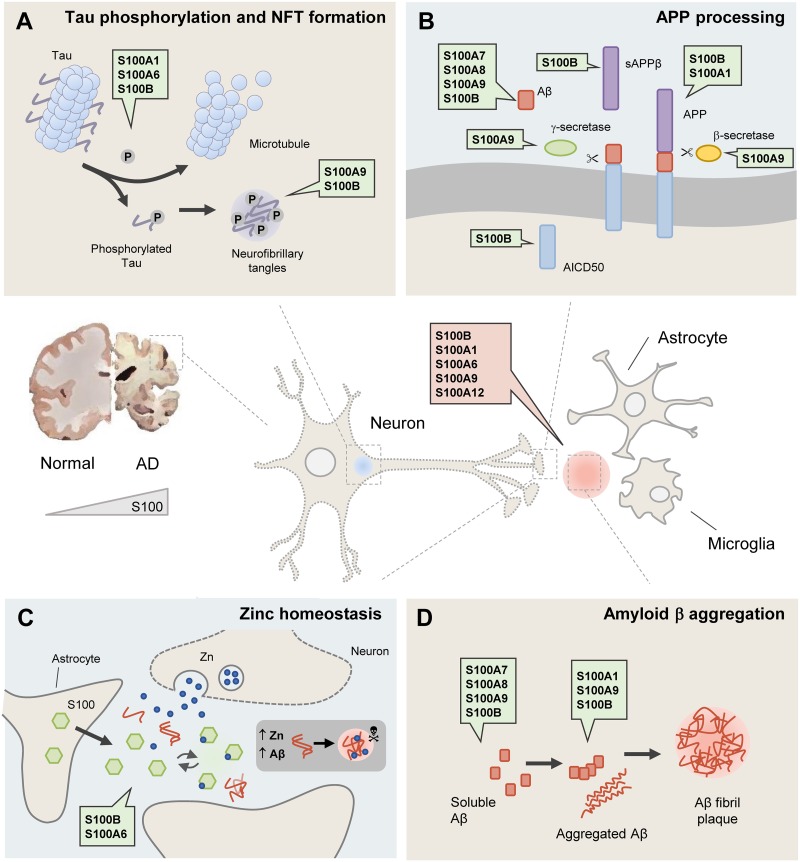
S100 proteins are involved in the main processes associated with Alzheimer’s disease (AD). In the AD brain, affected neurons (central panel) are damaged due to the formation of intracellular neurofibrillary tangles (represented by the blue dot) and extracellular amyloid species including an ensemble of low molecular weight aggregates, protofibrils and fibrils (represented by the red dot). As a result of astrocyte and microglia over-activation, some S100 proteins become upregulated, being implicated in several molecular processes altered in AD **(A–D)**. **(A)** Tau phosphorylation and NFT formation. S100A1, S100A6 and S100B are involved in the disassembly of microtubules and Tau release, while S100A9 and S100B are found within neurofibrillary tangles. **(B)** APP processing. Several S100 proteins are implicated in APP cleavage and its amyloidogenic processing. S100A9 regulates γ- and β-secretase expression and activity and S100B and S100A1 regulate APP levels. Moreover, S100A7, S100A8, S100A9 and S100B influence Aβ levels (Table 1). **(C)** Zinc homeostasis. Due to their zinc-binding properties, S100B and S100A6 have zinc-buffering activities that are related to neuroprotective roles; and S100A6 reduces zinc levels and senile plaque load in PS/APP mouse brains. **(D)** Amyloid β aggregation. S100A1, S100A9, and S100B proteins can interact, modulate the aggregation and co-aggregate with the Aβ peptide. Several S100 proteins (S100B, S100A1, S100A6, S100A8, S100A9, and S100A12), are found within amyloid plaques and in astrocytes and/or microglia around amyloid deposits. Further details and references can be found in the text and in Table 1.

Considering the involvement of S100 proteins in multiple regulatory functions in the brain, the fact that they have age- and damage- related expression, and a direct involvement in neuroinflammation, it is not surprising that they are implicated in molecular processes associated with AD pathogenesis (Figure 1).

Most of the available studies report essentially deleterious effects of S100 proteins in AD pathological processes. Indeed, elevated levels of S100 proteins around amyloid plaques and neurofibrillary tangles, exacerbate neuroinflammation and interfere with APP processing and with several AD-related proteins and signaling pathways. This scenario is compatible with an aggravating role as part of downstream inflammatory processes in later stages of AD neurodegeneration. However, some protective roles of S100 proteins are emerging as equally important. Indeed, it is now proposed that early inflammatory responses start at very early stages of the AD neurodegenerative process, most likely before amyloid plaques are formed (Cuello, 2017). This process would involve an elevation of inflammatory molecules, including S100, at the earliest stages of amyloid pathology, even before the onset of the disease phenotypes. Interestingly, several recent studies suggest that low levels of S100B protect cells against Aβ42-mediated cytotoxicity and that low levels of S100A9 inhibit Aβ42 cytotoxicity. This has led to the recent proposal of a new chaperone-like function for S100B (Cristovao et al., 2018), which seems to be extensible to a metal buffering activity (Hagmeyer et al., 2017), both of which are certainly relevant in the context of AD. Therefore, also in AD, S100 proteins may exert different functions according to their (extra)cellular concentrations. At early inflammatory stages and relatively low concentrations they play protective roles, while later in pathology, at higher concentrations, they play essentially disease aggravating roles. This is in line with prior evidence that suggests that decreasing the levels of S100 proteins may be a strategy to mitigate AD progression. The development of S100 neutralizing antibodies and small molecules is already a current therapeutic approach in cancer, autoimmune diseases and chronic inflammatory disorders, as reviewed in Bresnick (2018), and could present a promising strategy for AD as well. Ongoing research in our laboratory will shed light into this subject in the near future.

## Author Contributions

All authors listed have made a substantial, direct and intellectual contribution to the work, and approved it for publication.

## Conflict of Interest Statement

The authors declare that the research was conducted in the absence of any commercial or financial relationships that could be construed as a potential conflict of interest.
